# Real-world safety profile of alectinib: a 10-year pharmacovigilance study based on the FDA adverse event reporting system

**DOI:** 10.3389/fmed.2026.1894585

**Published:** 2026-08-27

**Authors:** Le Zhang, DanDan Li, ChenJing Luo, Li Fan, Min Hu, Fei Ye, Peng Gu

**Affiliations:** Department of Pharmacy, The Second Affiliated Hospital of Army Medical University, Chongqing, China

**Keywords:** adverse events, Alectinib, disproportionality analysis, FAERS database, pharmacovigilance

## Abstract

**Objective:**

Alectinib is the standard first-line therapy for treatment-naïve ALK-positive non-small cell lung cancer (NSCLC). Given its increasing clinical utilization, a comprehensive pharmacovigilance evaluation of its adverse events (AEs) is warranted.

**Methods:**

We assessed alectinib-associated AEs using the FDA Adverse Event Reporting System (FAERS) database from Q4 2015 to Q3 2025. AEs were categorized per MedDRA, and four disproportionality analysis algorithms were used to detect potential safety signals.

**Results:**

A total of 8,602 reports with alectinib as the primary suspect drug were included. Overall, 51.1% were serious adverse events (SAEs). Median AE onset was 120 days (IQR: 27–428), with 27.4% within 30 days and 28.9% after 360 days. All algorithms identified 108 preferred terms meeting positive safety signal criteria. Besides well-recognized AEs, novel signals emerged: hypertriglyceridemia, hypercholesterolemia, pericardial effusion, erythema multiforme, muscle fatigue, and myositis. Multivariate regression showed male sex and age ≥65 years were independently linked to higher SAE risk.

**Conclusion:**

Leveraging a large pharmacovigilance database, this study comprehensively characterized alectinib's safety profile. It confirmed established signals and uncovered novel potential AEs needing further prospective research. Subgroup disparities and key monitoring windows were identified, deepening mechanistic understanding and supporting optimized clinical risk mitigation strategies. It should be noted that disproportionality analysis is used to detect potential safety signals rather than establishing causal relationships, and all identified signals require further clinical evaluation.

## 1 Introduction

Lung cancer is one of the malignant tumors with the highest morbidity and mortality worldwide, among which non-small cell lung cancer (NSCLC) accounts for approximately 85% (1). Due to the insidious early symptoms, 41% of patients are diagnosed at locally advanced or metastatic stages (stage IIIB–IV) at initial presentation, with limited treatment options and extremely poor prognosis. In this context, driver gene-guided molecular targeted therapy has become a core strategy to improve the survival of patients with advanced NSCLC. Anaplastic lymphoma kinase (ALK) fusion is one of the key driver genes in NSCLC, with a positivity rate of approximately 9% in the Chinese population and 3%−7% globally. ALK-tyrosine kinase inhibitors (ALK-TKIs) effectively inhibit tumor cell proliferation and metastasis by specifically blocking the abnormal ALK phosphorylation signaling pathway (2, 3), and have thus become the standard treatment for ALK-positive advanced NSCLC.

The first approved ALK-TKI, crizotinib, which significantly prolonged progression-free survival (PFS) compared with conventional chemotherapy. However, almost all patients developed resistance after a median of 10.9 months of treatment, mainly attributed to secondary ALK mutations, activation of downstream signaling pathways, and insufficient central nervous system (CNS) penetration (4). Alectinib is a highly selective, CNS-active ALK inhibitor that not only effectively overcomes crizotinib-resistant mutations but also exhibits stronger inhibitory activity, and its metabolites possessing equivalent antitumor efficacy. The drug was first approved by the U.S. Food and Drug Administration (FDA) in December 2015 for the treatment of metastatic ALK-positive NSCLC with progression on or intolerance to crizotinib. Owing to its favorable clinical benefits, it was subsequently recommended for approval by the European Medicines Agency (EMA) in February 2017, followed by its launch in China in August 2018. Currently, alectinib has been widely adopted as a preferred standard first-line therapy for treatment-naïve ALK-positive NSCLC, as recommended in several national clinical practice guidelines (5–7).

Alectinib has shown significantly superior efficacy to crizotinib in a number of randomized phase III clinical trials. Preliminary results from the ALEX study demonstrated that alectinib significantly improved progression-free survival (PFS), reduced the risk of CNS progression, and exhibited a more favorable tolerability profile (8). The final PFS analysis confirmed that the median PFS was 34.8 months with alectinib, remarkably longer than the 10.9 months observed with crizotinib. Final mature overall survival (OS) data released in 2026 revealed that the median OS was 81.1 months in the alectinib group, with a 7-year OS rate of 48.6% and median duration of response (DOR) of 42.3 months, all of which were significantly superior to those in the crizotinib group (9). These findings were consistent with those from the ALESIA study in Asian patients and the J-ALEX study in Japanese patients (10, 11). The BFAST study (12), which used blood-based next-generation sequencing to identify targetable genomic alterations, further validated the efficacy of alectinib in treatment-naïve patients with advanced or metastatic non-small cell lung cancer. In the ALINA study (13), alectinib also demonstrated significantly superior efficacy compared with standard chemotherapy in the adjuvant setting. These high-level evidence-based data have firmly established alectinib as the standard first-line therapy for ALK-positive advanced NSCLC. However, its long-term clinical use necessitates rigorous evaluation of its safety and adverse reactions.

Current adverse reaction information listed in the package insert is primarily derived from randomized controlled trials (RCTs), including two phase II single-arm studies (NP28673 and NP28761) and two phase III trials (ALEX and ALINA) (8, 13, 14). Common adverse effects (AEs) include constipation, myalgia/arthralgia, peripheral or facial edema, elevated serum bilirubin and liver enzymes, anemia, and various types of rash, which are predominantly grade 1–2 in severity with a low discontinuation rate. However, RCTs are limited by small sample sizes, short follow-up durations, strict enrollment criteria, and good patient compliance, raising questions about the generalizability of their results to real-world populations with multiple comorbidities, long-term medication use, or frequent dose adjustments. Therefore, conducting real-world big data-based adverse reaction monitoring studies of alectinib is of great significance for supplementing existing safety evidence and guiding rational clinical medication. The U.S. FDA Adverse Event Reporting System (FAERS), as one of the largest global databases of adverse drug events, provides a valuable data resource for such real-world studies. This study aims to systematically analyze the characteristics of alectinib-related AEs based on the FAERS database using disproportionality analyses (ROR, PRR, BCPNN, MGPS) as signal detection tools, thus providing real-world evidence to support the safe clinical application of alectinib. This signal detection approach serves as an alternative method for identifying additional AEs not normally reported.

## 2 Methods

### 2.1 Data sources and processing

This study compiled AEs reports from the FAERS database, spanning from Q4 2015 to Q3 2025. The generic name “alectinib” and the brand name “Alecensa” were used as the search terms in DRUGNAME. All data extraction, transformation, and analytical procedures were executed in the R software (version 4.4.3). To address inherent duplicate reports in quarterly FAERS releases, we performed deduplication and prioritized the latest submissions. For reports with the same CASEID, the report with the latest FDA_DT value was retained. If reports had the same CASEID and FDA_DT, the report with the highest PRIMARYID was preserved.

**Figure 1:**
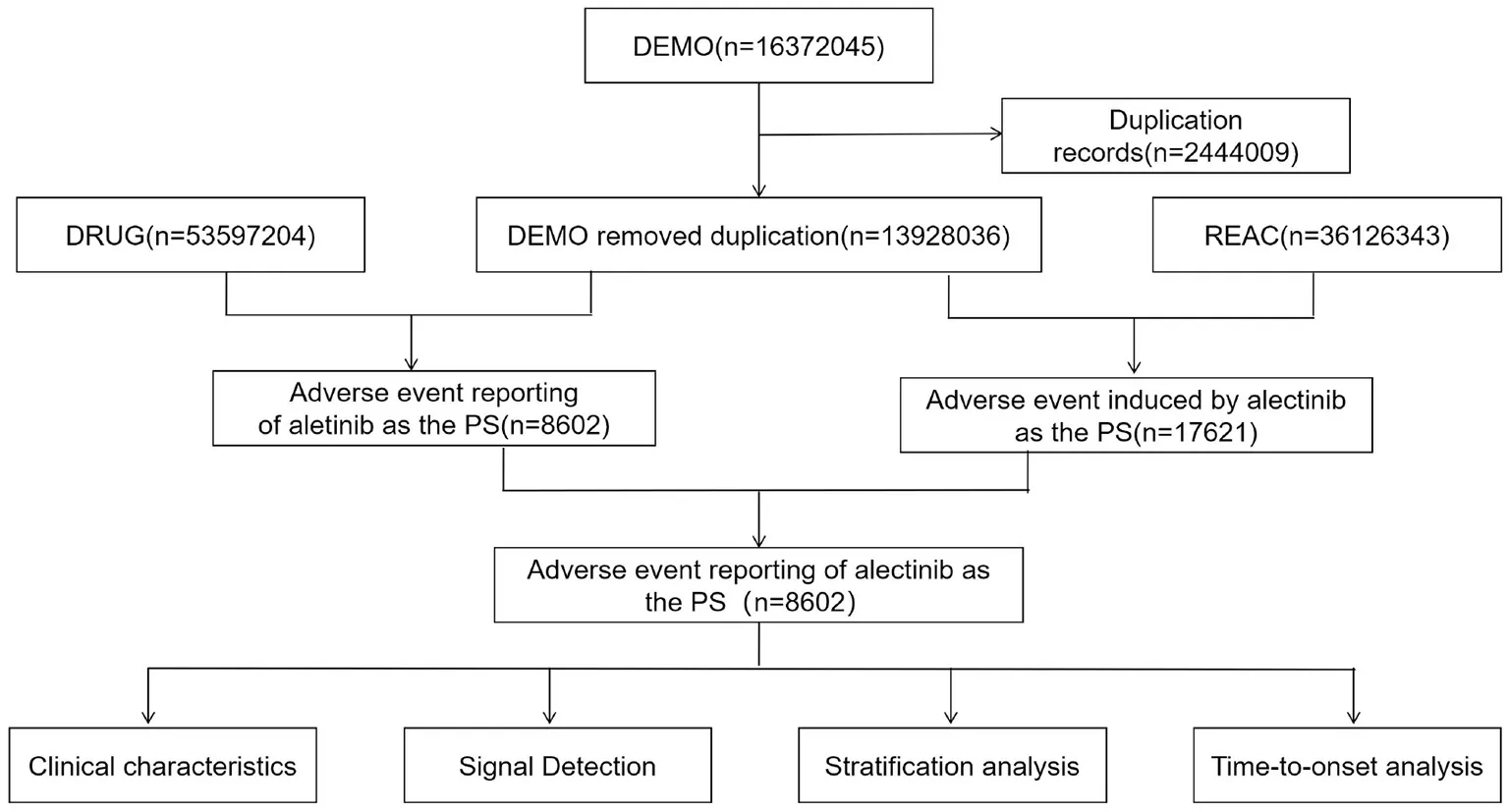
Flow-process diagram of alectinib-related AEs from the FAERS database.

Following deduplication, we retained only reports in which alectinib was designated the “primary suspect” drug for subsequent analysis. These reports were then extracted to construct our analytical dataset. All AEs were standardized and categorized using preferred terms (PT) and system organ classification (SOC) as defined by the Medical Dictionary for Regulatory Activities (MedDRA). Figure 1 illustrates the flowchart of obtaining alectinib-related AEs identified in the FAERS database.

### 2.2 Data analysis

We employed disproportionality analysis to assess potential associations between alectinib and AEs. Signal detection was performed using four quantitative pharmacovigilance algorithms: two frequentist methods [reporting odds ratio (ROR) and proportional reporting ratio (PRR)], which exhibit high sensitivity, and two Bayesian approaches [Bayesian confidence propagation neural network (BCPNN) and multi-item gamma Poisson shrinker (MGPS)], which enable robust handling of sparse data (15, 16). This multimethod approach enhances the robustness of the results, with signal strength directly proportional to the magnitude of each metric's output value. It is important to note that disproportionality analysis detects reporting imbalances rather than establishing causal relationships or statistical significance. A potential safety signal was defined as an AE term meeting the threshold of at least one algorithm. AE terms meeting all four algorithm criteria simultaneously were considered to exhibit stronger disproportionality, as agreement across multiple algorithms reduces method-specific bias, and thus prioritized for further clinical validation. Conversely, AE terms with metrics below the predefined thresholds were regarded as absence of potential signals, indicating no detectable reporting imbalance for these events in the database. The calculation details and threshold criteria for all algorithms are provided in Supplementary Table 1. During data cleaning, reports with unrelated indications, missing dose information, and off-label use were manually excluded.

The latency period of AEs was defined as the time interval from the initiation of drug administration (START_DT) to the onset of AEs (EVENT_DT). To guarantee data quality, all reports with chronological errors, specifically those where EVENT_DT was earlier than START_DT, or with inconsistent time-related documentation were systematically excluded from the subsequent analysis.

Additionally, to identify the disproportional AE signals between male and female patients, as well as between patients aged ≥65 years and those aged 18–64 years in alectinib-treated patients, we employed the formula of the ROR method. Forest plots were generated using the R package “ggplot2” (version 4.4.3) to visualize ROR values with 95% confidence intervals. When the ROR is >1, it suggests that female patients are more likely to report the specific AE than male patients, and patients aged 65 years or older are more likely to report the specific AE than those aged 18–64 years. Conversely, when the ROR is < 1, it suggests that male patients are more likely to report the specific AE than female patients, and those aged 18–64 years are more likely to report the specific AE than patients aged 65 years or older.

AEs that were life-threatening or resulted in hospitalization, disability, or death were classified as serious adverse events (SAEs). The proportion of SAEs was calculated by dividing the number of SAEs by the total AE count. Reports were stratified into SAE and non-SAE groups to assess the impact of age and gender on severe outcomes. To ensure analytical accuracy, reports with missing values for sex or age were excluded. Pearson's chi-square test was used to compare SAE incidence (17). Risk associations were quantified using odds ratios (OR) with 95% confidence intervals (CI). All analyses were performed in the R software (version 4.4.3). A P-value < 0.05 was considered statistically significant.

## 3 Results

### 3.1 Descriptive characteristics

In this study, a total of 13,928,036 original case reports were included from the FAERS database between Q4 2015 and Q3 2025. Alectinib was identified as the suspected drug in 8,602 documented cases. The comprehensive safety profile of alectinib-associated AEs is summarized in Table 1. Key demographic characteristics showed a female predominance (54.6% vs. 36.6% male), with most cases occurring in adults aged 18–64 years (35.1%), while patients aged 65–84 years accounted for 25.6% of the sample.

**Table 1:** Clinical characteristics of reports with alectinib from the FAERS database (Q4 2015 to Q3 2025).

Characteristics	Report numbers, *n*	Report proportion, %
Number of reports	8,602	
Gender		
Female	4,694	54.6
Male	3,145	36.6
Missing	763	8.9
Age (years)		
< 18	43	0.5
18 ≤ and < 5	3,021	35.1
65 ≤ and < 85	2,201	25.6
≥85	131	1.5
Missing	3,206	37.3
Reported countries (Top five)		
United States	4,388	51
Japan	711	8.3
China	666	7.7
Italy	248	2.9
France	208	2.4
Outcome		
Hospitalization	1,245	14.5
Death	970	11.3
Life-Threatening	79	0.9
Disability	37	0.4
Congenital Anomaly	1	0
Other	6,270	72.9
Reported person		
Consumer	3,833	44.6
Health Professional	718	8.3
Pharmacist	623	7.2
Physician	3,221	37.4
Missing	207	2.4
Serious or non-serious reports		
Serious	4,393	51.1
Non-serious	4,209	48.9

The top five reporting countries were the United States (51.0%), Japan (8.3%), China (7.7%), Italy (2.9%), and France (2.4%). With respect to reporter attribution, consumers contributed the largest proportion of reports at 44.6%, followed by physicians (37.4%), health professionals (8.3%), and pharmacists (7.2%). This distribution highlights substantial patient engagement in pharmacovigilance, and the combined proportion of medical practitioners (52.9%) supports the professional validity of the dataset.

In terms of clinical outcomes, “Other serious” events constituted the largest proportion (72.9%), followed by hospitalization (14.5%), mortality (11.3%), life-threatening conditions (0.9%), and permanent disability (0.4%). It should be noted that fatal outcomes may partially stem from the progression of underlying diseases rather than solely from drug-induced toxicity.

**Figure 2:**
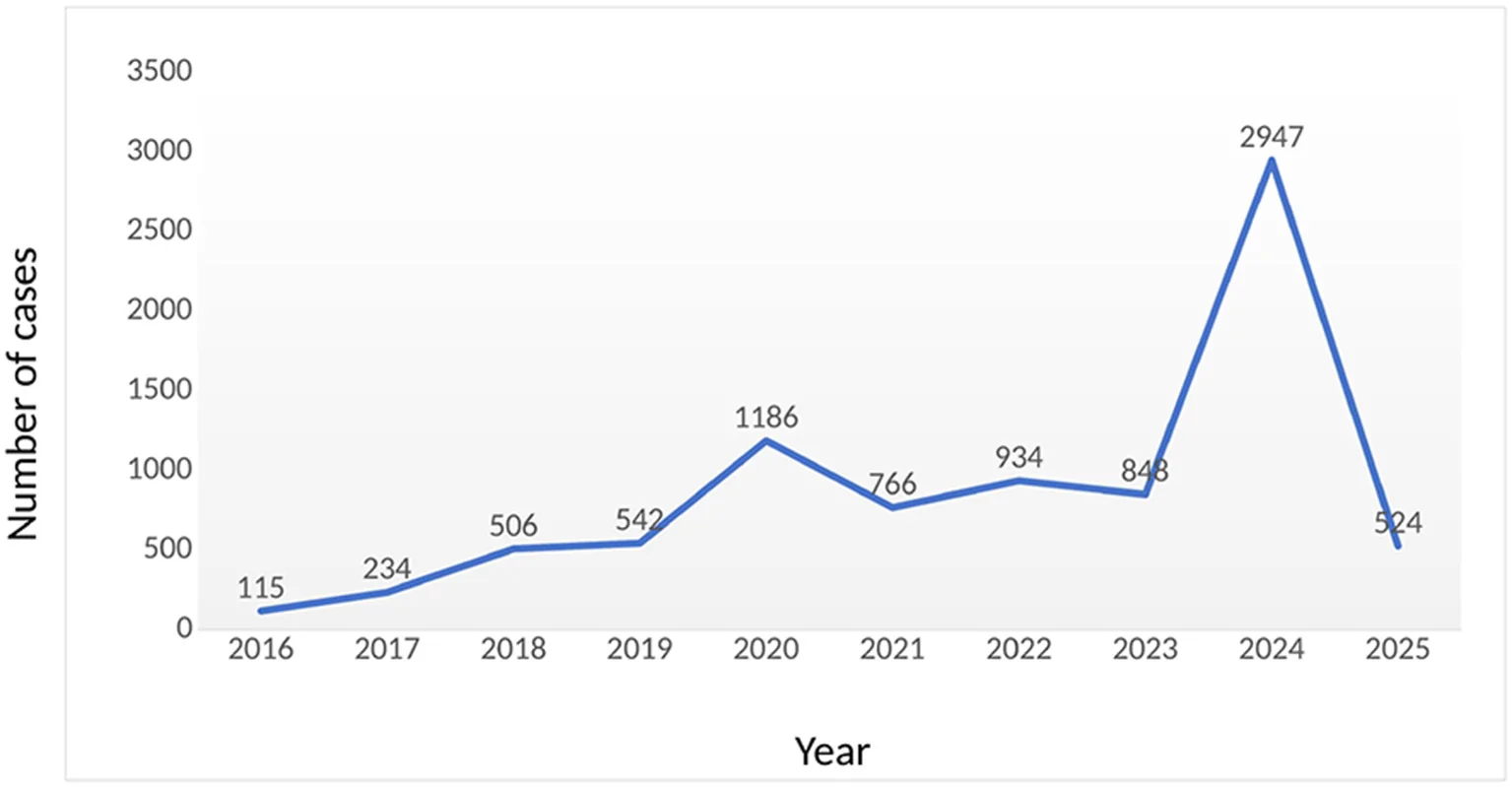
Distribution of AEs of alectinib from 2016 to the third quarter of 2025.

**Figure 3:**
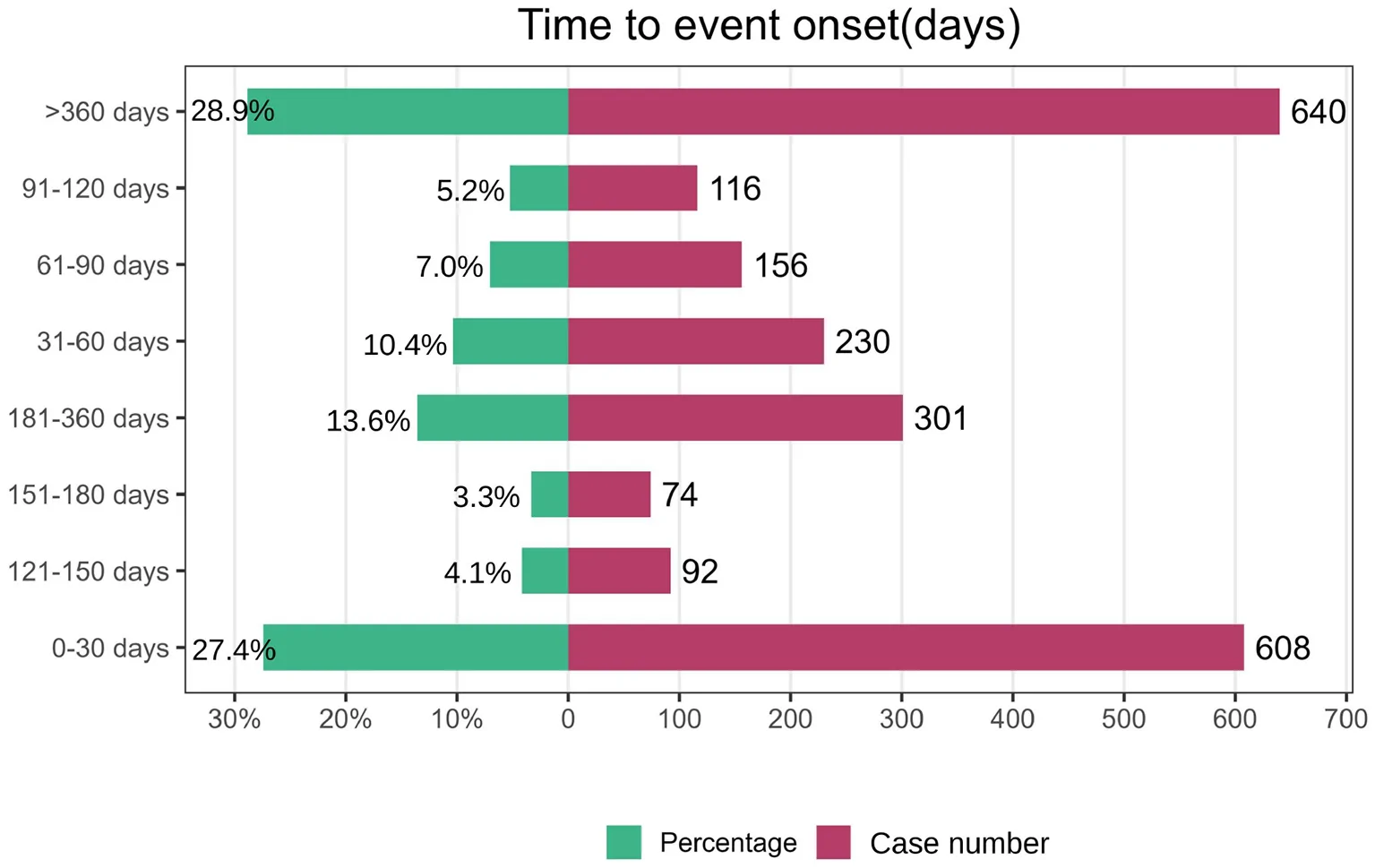
Time-to-onset of AEs.

Figure 2 also shows the temporal trends, indicating that the number of alectinib-associated AE reports reached a trough in 2016 (115 cases) and peaked in 2024 (2947 cases), with a steady upward trend from 2016 onward. The time-to-onset(TTO) analysis revealed a bimodal distribution of alectinib-associated AEs, with 27.4% occurring within 0–30 days and 28.9% occurring after more than 360 days of treatment (Figure 3).

### 3.2 Signals associated with SOC levels

SOC-level signal strengths are detailed in Supplementary Table 2. AEs involved 27 distinct organ systems. The most common SOC was General disorders and administration site conditions (*n* = 3,777, 28.0%), whereas the strongest signal was identified in Hepatobiliary disorders (*n* = 414, ROR 2.86, 95% CI: 2.59–3.15).

Disproportionality analysis identified 10 SOC-level potential signals (Table 2). Only Hepatobiliary disorders fulfilled all four predefined criteria simultaneously (ROR 2.86, PRR 2.82, EBGM 2.81, IC 1.49). The remaining 9 SOC-level potential signals met at least one criterion: Investigations, Blood and lymphatic system disorders, Neoplasms benign, malignant and unspecified, Respiratory, thoracic and mediastinal disorders, General disorders and administration site conditions, Metabolism and nutrition disorders, Cardiac disorders, Musculoskeletal and connective tissue disorders, and Gastrointestinal disorders. No potential signals were detected for Skin and subcutaneous tissue disorders, Renal and urinary disorders, or Infections and infestations, where the lower bound of the ROR 95% CI fell below 1.

**Table 2:** Adverse events of alectinib with positive signals detected by at least one disproportionality algorithm at the SOC level in the FAERS database.

System organ class (SOC)	Number of cases	ROR (95% Cl)	PRR (χ^2^)	EBGM (EBGM05)	IC (IC025)
Hepatobiliary disorders	414	2.86 (2.59–3.15)	2.82 (488.41)	2.81 (2.55)	1.49 (1.34)
Investigations	1,898	1.98 (1.89–2.08)	1.88 (826.37)	1.88 (1.79)	0.91 (0.84)
Blood and lymphatic system disorders	495	1.74 (1.59–1.9)	1.71 (149.78)	1.71 (1.57)	0.78 (0.64)
Neoplasms benign, malignant and unspecified (incl cysts and polyps)	792	1.58 (1.47–1.7)	1.56 (161.44)	1.55 (1.45)	0.64 (0.53)
Respiratory, thoracic and mediastinal disorders	1,223	1.55 (1.47–1.65)	1.52 (224.83)	1.52 (1.43)	0.6 (0.51)
General disorders and administration site conditions	3,777	1.26 (1.21–1.31)	1.2 (158.09)	1.2 (1.16)	0.27 (0.22)
Metabolism and nutrition disorders	417	1.17 (1.06–1.29)	1.16 (9.76)	1.16 (1.06)	0.22 (0.07)
Cardiac disorders	411	1.15 (1.04–1.27)	1.14 (7.69)	1.14 (1.04)	0.2 (0.05)
Musculoskeletal and connective tissue disorders	1,008	1.12 (1.05–1.2)	1.12 (13.03)	1.12 (1.05)	0.16 (0.07)
Gastrointestinal disorders	1,537	1.06 (1.01–1.12)	1.06 (4.93)	1.06 (1)	0.08 (0)

ROR, reporting odds ratio; CI, confidence interval; PRR, proportional reporting ratio; χ^2^, chi-squared; EBGM, empirical Bayesian geometric mean; EBGM05, the lower limit of the 95% CI of EBGM; IC, information component; IC025, the lower limit of the 95% CI of the IC.

### 3.3 Signal detection at the PT level

After excluding neoplasms and unspecified tumors, which may reflect disease progression rather than drug toxicity, 108 PTs met all four algorithm criteria simultaneously (Supplementary Table 3). Consistent with clinical trial data and prior pharmacovigilance findings, we identified signals for elevated transaminases, bradycardia, taste disturbance, haemolytic anemia, myalgia, and photosensitivity reaction. Notably, several unexpected AEs including hypertriglyceridaemia, hypercholesterolaemia, pericardial effusion, erythema multiforme, toxic epidermal necrolysis, and myositis were not listed in the drug label but exhibited strong disproportionality signals (Table 3). Conversely, AEs commonly reported in clinical trials such as nausea, vomiting, diarrhea, acute kidney injury, dizziness, visual disturbance, and photopsia did not meet our four-algorithm criteria.

**Table 3:** Novel and unexpected adverse events identified by four disproportionality algorithms for alectinib in the FAERS database.

System organ class (SOC)	Preferred term (PT)	Report number	ROR (95%Cl)	PRR (χ^2^)	EBGM (EBGM05)	IC (IC025)
Metabolism and nutrition disorders	Hypertriglyceridaemia	17	10.84 (6.73–17.46)	10.83 (150.88)	10.78 (6.69)	3.43 (2.12)
	Hypercholesterolaemia	17	7.84 (4.87–12.63)	7.83 (100.97)	7.81 (4.85)	2.96 (1.82)
	Hypoalbuminaemia	8	4.08 (2.04–8.17)	4.08 (18.57)	4.07 (2.04)	2.03 (0.64)
Ear and labyrinth disorders	Deafness unilateral	8	5.89 (2.94–11.8)	5.89 (32.41)	5.88 (2.94)	2.56 (0.97)
Infections and infestations	Tracheitis	3	14.01 (4.5–43.6)	14 (35.98)	13.92 (4.47)	3.8 (0.27)
	Appendicitis	10	3.93 (2.11–7.3)	3.93 (21.77)	3.92 (2.11)	1.97 (0.76)
	Diverticulitis	26	3.26 (2.22–4.79)	3.25 (40.55)	3.25 (2.21)	1.7 (1.03)
Investigations	Transferrin decreased	3	56.93 (18.08–179.32)	56.92 (160.36)	55.41 (17.59)	5.79 (0.45)
	Blood creatine phosphokinase decreased	3	29.42 (9.41–91.97)	29.41 (81.18)	29.01 (9.28)	4.86 (0.4)
	Myocardial necrosis marker increased	9	16.13 (8.37–31.08)	16.12 (126.66)	16 (8.3)	4 (1.76)
	Lipids increased	5	9.94 (4.13–23.94)	9.94 (40)	9.89 (4.11)	3.31 (0.81)
	Blood chloride increased	3	8.1 (2.61–25.18)	8.1 (18.6)	8.07 (2.6)	3.01 (0.1)
	Blood lactate dehydrogenase increased	24	7.26 (4.86–10.84)	7.25 (128.84)	7.23 (4.84)	2.85 (1.95)
	Protein total decreased	6	7.06 (3.17–15.74)	7.06 (31.09)	7.04 (3.16)	2.82 (0.82)
	Blood urea increased	16	5.28 (3.23–8.63)	5.28 (55.36)	5.27 (3.22)	2.4 (1.37)
	Haematocrit decreased	17	4 (2.48–6.43)	3.99 (38.09)	3.99 (2.48)	2 (1.09)
Nervous system disorders	Intracranial calcification	3	49.99 (15.9–157.15)	49.98 (140.57)	48.81 (15.53)	5.61 (0.45)
Injury, poisoning and procedural complications	Peripheral nerve injury	3	11.64 (3.74–36.23)	11.64 (29.02)	11.58 (3.72)	3.53 (0.22)
Musculoskeletal and connective tissue disorders	Muscle fatigue	19	16.09 (10.24–25.27)	16.07 (266.42)	15.95 (10.15)	4 (2.54)
	Myositis	11	4.78 (2.65–8.65)	4.78 (32.83)	4.77 (2.64)	2.25 (1.03)
Respiratory, thoracic and mediastinal disorders	Hydrothorax	11	43.13 (23.73–78.37)	43.1 (443.04)	42.23 (23.24)	5.4 (2.41)
	Pleural effusion	104	6.78 (5.59–8.23)	6.75 (508.22)	6.73 (5.55)	2.75 (2.39)
	Pulmonary oedema	61	5.36 (4.17–6.89)	5.34 (214.92)	5.33 (4.15)	2.41 (1.95)
Skin and subcutaneous tissue disorders	Erythema multiforme	16	7.35 (4.5–12.01)	7.34 (87.34)	7.32 (4.48)	2.87 (1.71)
	Skin wrinkling	5	6.26 (2.6–15.05)	6.26 (22.01)	6.24 (2.59)	2.64 (0.56)
	Onychoclasis	9	4 (2.08–7.69)	4 (20.19)	3.99 (2.08)	2 (0.71)
	Toxic epidermal necrolysis	14	3.68 (2.18–6.22)	3.68 (27.26)	3.67 (2.17)	1.88 (0.89)
Cardiac disorders	Pericardial effusion	46	7.69 (5.75–10.27)	7.67 (265.82)	7.64 (5.72)	2.93 (2.32)
	Atrioventricular block first degree	5	5.29 (2.2–12.73)	5.29 (17.35)	5.28 (2.19)	2.4 (0.44)
	Cardiac tamponade	6	4.77 (2.14–10.63)	4.77 (17.83)	4.76 (2.14)	2.25 (0.54)

ROR, reporting odds ratio; CI, confidence interval; PRR, proportional reporting ratio; χ^2^, chi-squared; EBGM, empirical Bayesian geometric mean; EBGM05, the lower limit of the 95% CI of EBGM; IC, information component; IC025, the lower limit of the 95% CI of the IC.

### 3.4 Subgroup analysis

**Figure 4:**
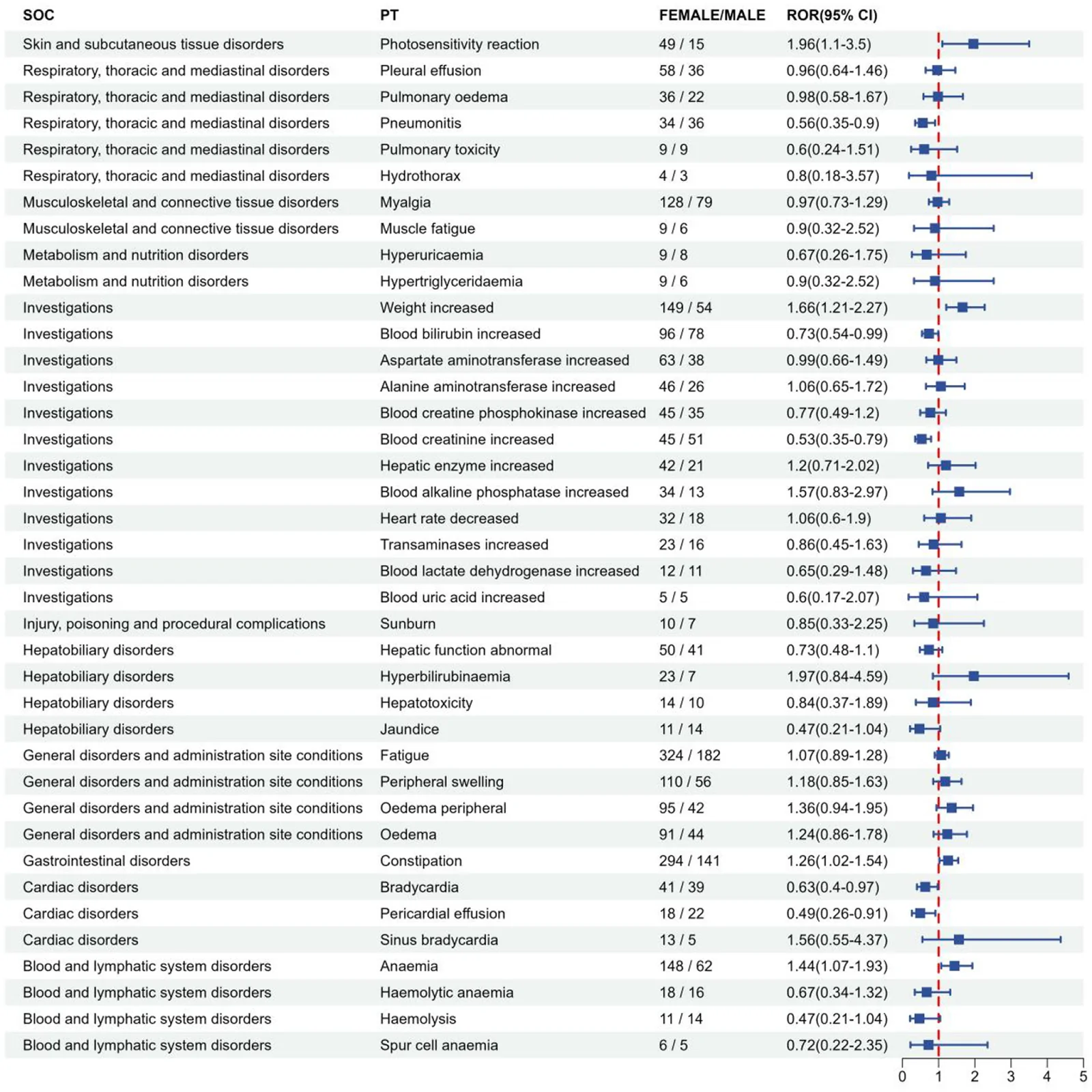
Gender subgroup analysis of alectinib-associated adverse events in the FAERS database, comparing adverse events occurrences between male and female patients. SOC, system organ class; PT, preferred term; ROR, reporting odds ratio; CI, confidence interval.

To evaluate sex-related differences, we identified 39 PTs with disproportionate reporting frequencies between male and female patients (Figure 4). AEs more frequently reported in males included pleural effusion, pulmonary oedema, pneumonitis, pulmonary toxicity, hydrothorax, myalgia, muscle fatigue, hyperuricaemia, hypertriglyceridaemia, hepatic enzyme abnormalities (elevated bilirubin, ALT, AST, CPK, LDH, transaminases), hyperuricaemia, hepatic dysfunction, hepatotoxicity, jaundice, pericardial effusion, and haemolytic events (haemolytic anemia, haemolysis, spur cell anemia). AEs more frequently reported in females included photosensitivity reaction, weight increased, hepatic enzyme abnormalities (elevated ALT, alkaline phosphatase), heart rate decreased, fatigue, peripheral oedema, constipation, sinus bradycardia, and anemia.

**Figure 5:**
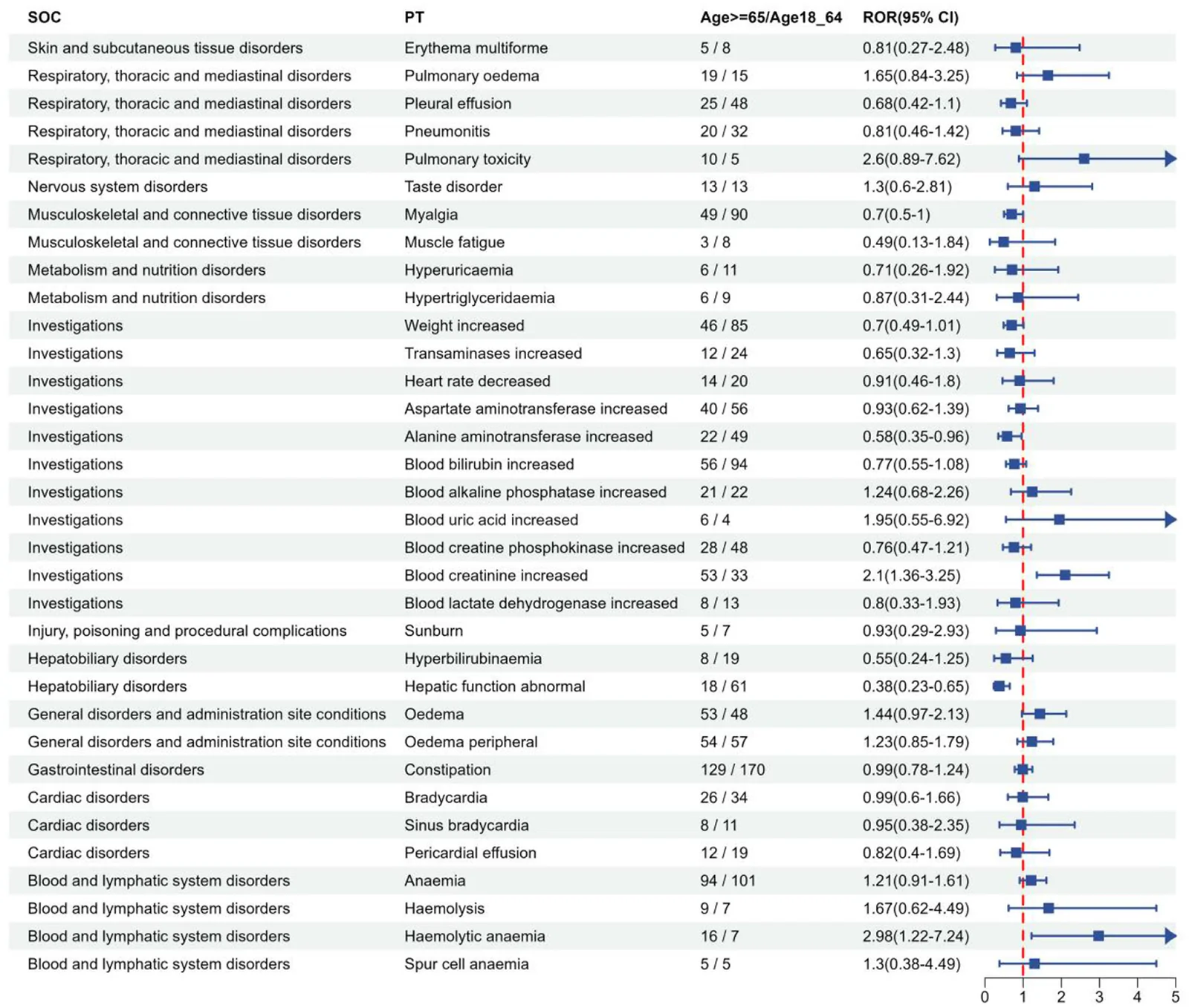
Age subgroup analysis of alectinib-associated adverse events in the FAERS database, comparing adverse events occurrences between patients aged 18–64 years and those aged ≥65years. SOC, system organ class; PT, preferred term; ROR, reporting odds ratio; CI, confidence interval.

For age-related differences, 34 PTs showed disproportionate reporting between patients aged 18–64 years and those aged ≥65 years (Figure 5). AEs more common in younger patients included erythema multiforme, pleural effusion, pneumonitis, myalgia, muscle fatigue, hyperuricaemia, hypertriglyceridaemia, weight increased, hepatic enzyme abnormalities (elevated transaminases, bilirubin, ALT, AST, CPK, LDH, hyperbilirubinaemia), hepatic dysfunction, constipation, bradycardia, sinus bradycardia, pericardial effusion, and sunburn. AEs more common in older patients included pulmonary oedema, pulmonary toxicity, taste disorder, elevated alkaline phosphatase and blood uric acid, elevated creatinine, oedema, and haemolytic events (anemia, haemolysis, haemolytic anemia, spur cell anemia).

SAEs were recorded in 4,393 reports (51.1%). Although women accounted for a higher proportion of SAE cases (56% vs. 44%), males exhibited a significantly higher likelihood of SAEs (OR = 1.35, 95% CI: 1.23–1.48, *P* < 0.001). Patients aged ≥65 years also demonstrated an increased risk of SAEs compared to those aged 18–64 years (65–74 years: OR = 1.57, 95% CI: 1.33–1.86; ≥75 years: OR = 1.56, 95% CI: 1.30–1.87; both *P* < 0.001) (Table 4). These findings suggest that both male gender and older age represent positive safety signals for alectinib-associated SAEs, warranting enhanced clinical monitoring in these populations.

**Table 4:** Comparison of gender and age distribution between serious and non-serious reports from the FAERS database.

Characteristic	Serious/Non-serious, *n*	*P*-value	OR (95% CI)
Gender			
Female(Reference)	2,267/2,427	*P* < 0.001	–
Male	1,752/1,393		1.35 (1.23–1.48)
Age group, years			
18–64 (Reference)	1,491/1,530	–	–
65–74	437/285	*P* < 0.001	1.57 (1.33–1.86)
≥75	340/224	*P* < 0.001	1.56 (1.3–1.87)

## 4 Discussion

Alectinib has been widely used in treating ALK-positive NSCLC for over a decade. Current pharmacovigilance studies of alectinib based on the FAERS database mostly focus on comparisons with other ALK-TKIs or analyses of single AEs such as pleural effusion, epilepsy, arrhythmia, and hepatotoxicity and lack a comprehensive safety evaluation of this agent. Therefore, this study was designed to characterize the full AE profile of alectinib.

In this study, females accounted for 54.6% of all alectinib-related AE reports, which is consistent with the epidemiological characteristic that ALK rearrangement positivity is more prevalent in non-smoking female patients with NSCLC (18). Notably, the number of reports peaked in 2024, accounting for 34.3% of the total. This sharp increase may be mainly attributed to the recent approval of alectinib as adjuvant therapy for ALK-positive NSCLC, which greatly expanded the target population and strengthened post-marketing safety monitoring and AE reporting. Additionally, the TTO analysis revealed a distinct bimodal distribution, with notable proportions occurring in both the early (0–30 days, 27.4%) and late (>360 days, 28.9%) phases of treatment and a median onset time of 120 days. This bimodal pattern is consistent with the pharmacological properties of alectinib as a long-term targeted therapy for ALK-positive NSCLC. Early-onset AEs may be associated with acute pharmacological effects of the drug, while late-onset events are likely related to cumulative drug exposure over prolonged treatment courses. This finding underscores the necessity of individualized safety monitoring throughout the entire treatment period, including close surveillance in the early phase to detect acute reactions and continuous long-term follow-up to identify delayed AEs.

At the SOC level, hepatic disorders represented a positive signal that simultaneously met the criteria of all four disproportionality analysis algorithms. This finding is consistent with previous clinical trial results (8, 19), confirming that alectinib is associated with definite and potentially severe hepatotoxicity. Alectinib-induced hepatotoxicity manifests as elevated liver enzymes, including alanine aminotransferase (ALT) and aspartate aminotransferase (AST), as well as hyperbilirubinemia, which may progress to severe liver injury with histological features such as focal necrosis and bile stasis (20). The occurrence of alectinib-induced hyperbilirubinemia was up to 36.2% at all grades in a clinical trial, 3 and the incidence of grade 3 (5–10 times the upper limit of normal) or higher elevation is about 2%. Regarding its mechanism, the injury involves mitochondrial damage-induced oxidative stress and pyroptosis accompanied by suppressed Nrf2/HO-1 antioxidant signaling that exacerbates hepatic damage (21), thus warranting close liver function monitoring during treatment.

Metastasis is a key factor affecting the prognosis and therapeutic strategy of NSCLC. Some organs are more prone to tumor metastasis, with common sites including the brain, bone, adrenal glands, and lymph nodes (22, 23). Tumor involvement of the recurrent laryngeal nerve may also lead to vocal cord paralysis (22). At the SOC level, malignant tumor-related categories, including lung neoplasm malignancy, metastases to central nervous system, metastases to bone, are mainly associated with disease progression. At the PT level, events such as elevated carcinoembryonic antigen, death, lymphangitis, brain disorders, bone lesions, and malignant pleural effusion are more likely to be related to disease progression rather than adverse drug reactions.

For the metabolic system, alectinib may induce hyperuricemia, a validated safety signal and labeled adverse reaction, as well as hypertriglyceridemia and hypercholesterolemia, two potential emerging safety signals that requiring clinical attention. Among ALK-TKIs, dyslipidemia is most prevalent with lorlatinib, while alectinib-induced lipid abnormalities are relatively rare (24), though severe hypertriglyceridemia can occur in susceptible populations (25). Its direct lipid metabolic targets remain unclear, but indirect pathways include exacerbating triglyceride-rich lipoprotein excess, impairing lipoprotein lipase function or upregulating apolipoprotein C-III, and inducing fat distribution disorders to promote metabolic syndrome (26). These disturbances also increase the risks of diabetes and cardiovascular disease (27, 28). Enhanced metabolic surveillance and regular assessments of lipid, glucose, weight and waist circumference are recommended. If dietary interventions or anti-obesity medications are required, interdisciplinary collaboration is advised to avoid interference with alectinib absorption (29).

In terms of skin toxicity, this study identified signals of erythema multiforme and toxic epidermal necrolysis associated with alectinib. Severe Grade 3 skin rashes caused by alectinib treatment are rare events (0%−3%) (8). Currently, only a few sporadic cases of alectinib-related erythema multiforme have been reported (30–32). Some of these cases were confounded by sequential treatment with immune checkpoint inhibitors, which is not consistent with the standard first-line strategy recommended by current guidelines (32). The causal relationship between alectinib and erythema multiforme therefore requires further validation in large-scale studies. In addition, there is no direct evidence to support a link between alectinib and toxic epidermal necrolysis. The signal detected in this study may be related to inherent biases in spontaneous reporting systems. Close monitoring of skin reactions is still recommended during clinical treatment with alectinib.

This study demonstrates that alectinib-associated cardiotoxicity presents with two core phenotypes: sinus bradycardia and pericardial effusion. Sinus bradycardia occurs in 40%−50% of patients, mostly asymptomatic, with only a minority requiring dose reduction or discontinuation (33). Events typically onset within 2–3 months of treatment, with higher risk in patients with a fast baseline heart rate or comorbid hypertension. This adverse reaction is concentration-dependent and reversible, with no impact on QT interval or left ventricular function (34). Mechanistically, mouse sinoatrial node RNA-seq and patch-clamp experiments confirm selective downregulation of Cav1.3 channel expression and current density, slowing diastolic depolarization and producing a “pure sinus” bradycardic phenotype (35), but the exact pathway in the human sinoatrial node remains to be verified. On the other hand, our study identified a novel safety signal of alectinib-related pericardial effusion, a finding not documented in the current package insert. Notably, this adverse event exhibits a bidirectional effect: alectinib may induce benign pericardial effusion via off-target effects, inflammatory responses and endothelial permeability abnormalities (36), while preclinical and clinical evidence also confirm its ability to alleviate malignant pericardial effusion by controlling tumor burden. Such drug-induced effusions resolve upon discontinuation, and other etiologies such as tumor progression must first be excluded clinically. Additionally, the UK Yellow Card System indicates alectinib has the strongest overall cardiovascular AE signal among NSCLC therapies, most notably for sinus bradycardia, whereas other agents more commonly cause hypertension and related disorders. However, real-world data suggest alectinib has better cardiovascular safety than lorlatinib and brigatinib, with female-specific left ventricular failure signals reported but limited prospective evidence for systolic dysfunction (37). In summary, alectinib exhibits a unique cardiotoxicity profile dominated by sinus bradycardia, accompanied by pericardial effusion and rare left ventricular failure. Baseline cardiovascular assessment, early monitoring of heart rate and effusion imaging, attention to comorbidities, and multidisciplinary cardio-oncology follow-up are critical for balancing the safety and antitumor efficacy.

Alectinib is associated with anemia in a substantial proportion of patients, with a reported incidence as high as 47.2% in the ALESIA study (13, 19, 38). Such anemia is frequently accompanied by hemolysis characterized by abnormal red blood cell morphology, including acanthocytosis and stomatoacanthocytosis. These morphological changes result primarily from direct disruption of erythrocyte membrane band 3 protein and cytoskeletal complexes, as well as altered membrane lipid composition (38), rather than immune-mediated destruction. The abnormally shaped erythrocytes are sequestered and phagocytosed by the splenic mononuclear phagocyte system, which is consistent with an extravascular hemolytic pattern. These abnormalities can be significantly ameliorated by switching to alternative ALK-TKIs such as brigatinib (39). By contrast, immune hemolysis is rare, with only occasional antiglobulin test (DAT)-positive cases reported (40, 41), and may respond to dose reduction or treatment discontinuation. For clinical management, regular monitoring of erythrocyte morphology, hemolysis-related biomarkers and complete blood count is recommended. Asymptomatic or mild subclinical hemolysis can be managed with close observation alone, while dose reduction, temporary treatment discontinuation or switch to brigatinib/lorlatinib should be considered for patients with significant or symptomatic hemolysis.

Although FAERS represents the largest publicly available spontaneous reporting database for postmarketing pharmacovigilance, its inherent limitations should be acknowledged. First, the database relies on a voluntary reporting system, leading to nonsystematic data collection and substantial underreporting. As FAERS does not collect reliable denominator data (i.e., total number of patients exposed), true incidence rates cannot be calculated. Although dose information is present in some reports, dose-specific AE frequency analysis is not feasible due to inconsistent dose documentation across reports and inability to determine the total exposed population at each dose level. Second, FAERS is a retrospective database inherently associated with methodological biases, including variable report quality and completeness. Missing baseline characteristics, incomplete documentation of concomitant medications, and regional reporting biases may interfere with the accurate interpretation of AE signals and complicate the adjustment for confounding factors. Third, this study employed disproportionality analyses (ROR, PRR, BCPNN, MGPS) as signal detection tools to identify potential safety signals, not to establish causal relationships. Well-established causality assessment methods (e.g., WHO-UMC, Naranjo algorithm) require detailed individual case information that FAERS structurally cannot provide. Our findings only indicate statistical associations between alectinib exposure and AEs. This is due to high heterogeneity, the inability of disproportionality analysis to fully eliminate confounding from concomitant drugs, and the lack of longitudinal follow-up information to verify the temporality and persistence of exposure-response relationships.

## 5 Conclusion

Using the FAERS database, this study systematically evaluated alectinib-associated adverse events (AEs), which affect multiple organ systems with gender and age subgroup differences. We confirmed expected AEs including hepatotoxicity, hyperuricaemia, haemolytic anemia, bradycardia, and photosensitivity, while identifying novel signals such as hypertriglyceridaemia, hypercholesterolaemia, pericardial effusion, erythema multiforme, muscle fatigue, and myositis. These findings corroborate clinical trial observations, reveal underappreciated risks requiring enhanced monitoring (particularly in the first month and after 360 days of treatment), and highlight the need for mechanistic and prospective studies to optimize risk mitigation strategies for this ALK-TKI.

## Data Availability

Publicly available datasets were analyzed in this study. Data were obtained from the publicly available FDA Adverse Event Reporting System (FAERS) FDA Adverse Event Reporting System Database. No new datasets were generated.
