# Supplementary material for: Real-world safety profile of alectinib: a 10-year pharmacovigilance study based on the FDA adverse event reporting system
Source: Front Med (Lausanne). 2026 Aug 27;13:1894585. doi: 10.3389/fmed.2026.1894585 (PMC13557985; doi:10.3389/fmed.2026.1894585)
Supplement: Supplementary file 1 [file Table_1.docx]

**Supplementary Table 1.** Four major algorithms used for signal detection.

| Algorithms | Equation | Criteria |
| --- | --- | --- |
| ROR | ROR=ad/b/c | lower limit of 95% CI>1, N≥3 |
|  | 95%CI=e^ln(ROR)±1.96(1/a+1/b+1/c+1/d)^0.5^ |  |
| PRR | PRR=a(c+d)/c/(a+b) | PRR≥2, χ^2^≥4, N≥3 |
|  | χ^2^=[(ad-bc)^2](a+b+c+d)/[(a+b)(c+d)(a+c)(b+d)] |  |
| BCPNN | IC=log_2_a(a+b+c+d)(a+c)(a+b) | IC025>0 |
|  | 95%CI= E(IC) ± 2V(IC)^0.5 |  |
| MGPS | EBGM=a(a+b+c+d)/(a+c)/(a+b) | EBGM05>2 |
|  | 95%CI=e^ln(EBGM)±1.96(1/a+1/b+1/c+1/d)^0.5^ |  |

Abbreviation: ROR, reporting odds ratio; PRR, proportional reporting ratio; BCPNN, bayesian confidence propagation neural network; MGPS, multi-item gamma Passion shrinker. 95%CI, 95% confidence interval; N, the number of reports; χ2, chi-squared; IC, information component; IC025, the lower limit of 95% CI of the IC; E(IC), the IC expectations; V(IC), the variance of IC; EBGM, empirical Bayesian geometric mean; EBGM05, the lower limit of 95% CI of EBGM.

Equation: a, number of reports containing both the target drug and target adverse drug reaction; b, number of reports containing other adverse drug reaction of the target drug; c, number of reports containing the target adverse drug reaction of other drugs; d, number of reports containing other drugs and other adverse drug reactions.

**Supplementary Table 2** Signal strength of adverse events with alectinib at SOC level from the FAERS database

| **System Organ Class(SOC)** | **number of Cases** | **ROR(95%Cl)** | **PRR(χ²)** | **EBGM**  **(EBGM05)** | **IC(IC025)** |
| --- | --- | --- | --- | --- | --- |
| Hepatobiliary disorders* | 414 | 2.86 ( 2.59 - 3.15 ) | 2.82 ( 488.41 ) | 2.81 ( 2.55 ) | 1.49 ( 1.34 ) |
| Investigations* | 1898 | 1.98 ( 1.89 - 2.08 ) | 1.88 ( 826.37 ) | 1.88 ( 1.79 ) | 0.91 ( 0.84 ) |
| Blood and lymphatic system disorders* | 495 | 1.74 ( 1.59 - 1.9 ) | 1.71 ( 149.78 ) | 1.71 ( 1.57 ) | 0.78 ( 0.64 ) |
| Neoplasms benign, malignant and unspecified (incl cysts and polyps)* | 792 | 1.58 ( 1.47 - 1.7 ) | 1.56 ( 161.44 ) | 1.55 ( 1.45 ) | 0.64 ( 0.53 ) |
| Respiratory, thoracic and mediastinal disorders* | 1223 | 1.55 ( 1.47 - 1.65 ) | 1.52 ( 224.83 ) | 1.52 ( 1.43 ) | 0.6 ( 0.51 ) |
| General disorders and administration site conditions* | 3777 | 1.26 ( 1.21 - 1.31 ) | 1.2 ( 158.09 ) | 1.2 ( 1.16 ) | 0.27 ( 0.22 ) |
| Metabolism and nutrition disorders* | 417 | 1.17 ( 1.06 - 1.29 ) | 1.16 ( 9.76 ) | 1.16 ( 1.06 ) | 0.22 ( 0.07 ) |
| Cardiac disorders* | 411 | 1.15 ( 1.04 - 1.27 ) | 1.14 ( 7.69 ) | 1.14 ( 1.04 ) | 0.2 ( 0.05 ) |
| Musculoskeletal and connective tissue disorders* | 1008 | 1.12 ( 1.05 - 1.2 ) | 1.12 ( 13.03 ) | 1.12 ( 1.05 ) | 0.16 ( 0.07 ) |
| Gastrointestinal disorders* | 1537 | 1.06 ( 1.01 - 1.12 ) | 1.06 ( 4.93 ) | 1.06 ( 1 ) | 0.08 ( 0 ) |
| Ear and labyrinth disorders | 78 | 1.02 ( 0.82 - 1.28 ) | 1.02 ( 0.04 ) | 1.02 ( 0.82 ) | 0.03 ( -0.29 ) |
| Skin and subcutaneous tissue disorders | 948 | 0.92 ( 0.86 - 0.99 ) | 0.93 ( 5.74 ) | 0.93 ( 0.87 ) | -0.11 ( -0.21 ) |
| Renal and urinary disorders | 298 | 0.9 ( 0.8 - 1.01 ) | 0.9 ( 3.31 ) | 0.9 ( 0.8 ) | -0.15 ( -0.32 ) |
| Infections and infestations | 845 | 0.88 ( 0.82 - 0.94 ) | 0.88 ( 14.34 ) | 0.88 ( 0.82 ) | -0.18 ( -0.28 ) |
| Nervous system disorders | 1190 | 0.87 ( 0.82 - 0.92 ) | 0.88 ( 21.2 ) | 0.88 ( 0.83 ) | -0.18 ( -0.27 ) |
| Endocrine disorders | 35 | 0.75 ( 0.54 - 1.04 ) | 0.75 ( 2.97 ) | 0.75 ( 0.54 ) | -0.42 ( -0.89 ) |
| Vascular disorders | 217 | 0.64 ( 0.56 - 0.73 ) | 0.65 ( 43.05 ) | 0.65 ( 0.56 ) | -0.63 ( -0.83 ) |
| Eye disorders | 194 | 0.55 ( 0.48 - 0.64 ) | 0.56 ( 68.44 ) | 0.56 ( 0.49 ) | -0.84 ( -1.04 ) |
| Congenital, familial and genetic disorders | 24 | 0.53 ( 0.35 - 0.78 ) | 0.53 ( 10.25 ) | 0.53 ( 0.35 ) | -0.93 ( -1.48 ) |

**Supplementary Table 2** (continued)

| **System Organ Class(SOC)** | **number of cases** | **ROR(95%Cl)** | **PRR(χ²)** | **EBGM**  **(EBGM05)** | **IC(IC025)** |
| --- | --- | --- | --- | --- | --- |
| Injury, poisoning and procedural complications | 1167 | 0.52 ( 0.49 - 0.55 ) | 0.55 ( 494.28 ) | 0.55 ( 0.52 ) | -0.87 ( -0.95 ) |
| Surgical and medical procedures | 131 | 0.51 ( 0.43 - 0.6 ) | 0.51 ( 61.5 ) | 0.51 ( 0.43 ) | -0.96 ( -1.21 ) |
| Reproductive system and breast disorders | 51 | 0.39 ( 0.29 - 0.51 ) | 0.39 ( 49.86 ) | 0.39 ( 0.29 ) | -1.37 ( -1.75 ) |
| Social circumstances | 32 | 0.39 ( 0.28 - 0.55 ) | 0.39 ( 30.4 ) | 0.39 ( 0.28 ) | -1.35 ( -1.83 ) |
| Psychiatric disorders | 341 | 0.36 ( 0.32 - 0.4 ) | 0.37 ( 390.92 ) | 0.37 ( 0.33 ) | -1.44 ( -1.6 ) |
| Immune system disorders | 50 | 0.23 ( 0.18 - 0.31 ) | 0.24 ( 125.14 ) | 0.24 ( 0.18 ) | -2.08 ( -2.47 ) |
| Product issues | 40 | 0.12 ( 0.09 - 0.16 ) | 0.12 ( 255.24 ) | 0.12 ( 0.09 ) | -3.02 ( -3.44 ) |
| Pregnancy, puerperium and perinatal conditions | 8 | 0.12 ( 0.06 - 0.24 ) | 0.12 ( 51.38 ) | 0.12 ( 0.06 ) | -3.05 ( -3.86 ) |

Abbreviation: ROR, reporting odds ratio; CI, confidence interval; PRR, proportional reporting ratio; **χ²**, chi-squared; EBGM, empirical Bayesian geometric mean; EBGM05, the lower limit of the 95% CI of EBGM; IC, information component; IC025, the lower limit of the 95% CI of the IC. * indicate a positive signal defined as at least one of the four algorithms that meet the criteria.

| **Supplementary Table 3** Signal strength of adverse events with alectinib at PT level from the FAERS database | | | | | | |
| --- | --- | --- | --- | --- | --- | --- |
| System Organ Class(SOC) | Preferred term(PT) | Report number | ROR(95%Cl) | PRR(χ²) | EBGM(EBGM05) | IC(IC025) |
| Metabolism and nutrition disorders | Hyperuricaemia | 18 | 17.7 ( 11.13 - 28.16 ) | 17.68 ( 280.89 ) | 17.54 ( 11.03 ) | 4.13 ( 2.56 ) |
|  | Hypertriglyceridaemia* | 17 | 10.84 ( 6.73 - 17.46 ) | 10.83 ( 150.88 ) | 10.78 ( 6.69 ) | 3.43 ( 2.12 ) |
|  | Hypercholesterolaemia* | 17 | 7.84 ( 4.87 - 12.63 ) | 7.83 ( 100.97 ) | 7.81 ( 4.85 ) | 2.96 ( 1.82 ) |
|  | Hypoalbuminaemia* | 8 | 4.08 ( 2.04 - 8.17 ) | 4.08 ( 18.57 ) | 4.07 ( 2.04 ) | 2.03 ( 0.64 ) |
|  | Fluid retention | 58 | 4.06 ( 3.13 - 5.25 ) | 4.05 ( 132.8 ) | 4.04 ( 3.12 ) | 2.01 ( 1.56 ) |
| Ear and labyrinth disorders | Deafness unilateral* | 8 | 5.89 ( 2.94 - 11.8 ) | 5.89 ( 32.41 ) | 5.88 ( 2.94 ) | 2.56 ( 0.97 ) |
| Hepatobiliary disorders | Hyperbilirubinaemia | 36 | 12.96 ( 9.34 - 17.99 ) | 12.94 ( 394.11 ) | 12.86 ( 9.27 ) | 3.69 ( 2.81 ) |
|  | Hepatic function abnormal | 96 | 9.87 ( 8.07 - 12.06 ) | 9.82 ( 757 ) | 9.77 ( 7.99 ) | 3.29 ( 2.87 ) |
|  | Ocular icterus | 9 | 9.46 ( 4.91 - 18.22 ) | 9.46 ( 67.76 ) | 9.42 ( 4.89 ) | 3.24 ( 1.44 ) |
|  | Hypertransaminasaemia | 15 | 6.54 ( 3.94 - 10.86 ) | 6.54 ( 70.14 ) | 6.52 ( 3.93 ) | 2.7 ( 1.55 ) |
|  | Hepatotoxicity | 31 | 4.86 ( 3.41 - 6.91 ) | 4.85 ( 94.62 ) | 4.84 ( 3.4 ) | 2.28 ( 1.6 ) |
|  | Jaundice | 26 | 4.59 ( 3.13 - 6.75 ) | 4.59 ( 72.84 ) | 4.58 ( 3.12 ) | 2.2 ( 1.46 ) |
|  | Liver disorder | 46 | 3.99 ( 2.99 - 5.33 ) | 3.98 ( 102.55 ) | 3.98 ( 2.98 ) | 1.99 ( 1.48 ) |
|  | Hepatitis | 23 | 3.62 ( 2.4 - 5.45 ) | 3.62 ( 43.47 ) | 3.61 ( 2.4 ) | 1.85 ( 1.11 ) |
| Infections and infestations | Lymphangitis✝ | 3 | 14.54 ( 4.67 - 45.25 ) | 14.53 ( 37.54 ) | 14.44 ( 4.64 ) | 3.85 ( 0.28 ) |
|  | Tracheitis* | 3 | 14.01 ( 4.5 - 43.6 ) | 14 ( 35.98 ) | 13.92 ( 4.47 ) | 3.8 ( 0.27 ) |
|  | Appendicitis* | 10 | 3.93 ( 2.11 - 7.3 ) | 3.93 ( 21.77 ) | 3.92 ( 2.11 ) | 1.97 ( 0.76 ) |
|  | Diverticulitis* | 26 | 3.26 ( 2.22 - 4.79 ) | 3.25 ( 40.55 ) | 3.25 ( 2.21 ) | 1.7 ( 1.03 ) |
| Investigations | Red blood cell acanthocytes present | 6 | 1366.59 ( 486.36 - 3839.85 ) | 1366.12 ( 4910.85 ) | 820.07 ( 291.86 ) | 9.68 ( 1.49 ) |
|  | Red blood cell morphology abnormal | 3 | 77.83 ( 24.57 - 246.54 ) | 77.82 ( 219.17 ) | 75.01 ( 23.68 ) | 6.23 ( 0.47 ) |
|  | Transferrin decreased* | 3 | 56.93 ( 18.08 - 179.32 ) | 56.92 ( 160.36 ) | 55.41 ( 17.59 ) | 5.79 ( 0.45 ) |
|  | Blood bilirubin unconjugated increased | 6 | 40.46 ( 18.03 - 90.78 ) | 40.44 ( 226.35 ) | 39.68 ( 17.68 ) | 5.31 ( 1.5 ) |
|  | Blood bilirubin increased | 190 | 32.18 ( 27.86 - 37.17 ) | 31.85 ( 5591.7 ) | 31.37 ( 27.16 ) | 4.97 ( 4.55 ) |
| **Supplementary Table 3 (continued)** | | | | | | |
| System Organ Class(SOC) | Preferred term(PT) | Report number | ROR(95%Cl) | PRR(χ²) | EBGM(EBGM05) | IC(IC025) |
| Investigations | Blood creatine phosphokinase decreased* | 3 | 29.42 ( 9.41 - 91.97 ) | 29.41 ( 81.18 ) | 29.01 ( 9.28 ) | 4.86 ( 0.4 ) |
|  | Blood creatine phosphokinase increased | 103 | 18.1 ( 14.9 - 21.98 ) | 18 ( 1639.38 ) | 17.85 ( 14.69 ) | 4.16 ( 3.66 ) |
|  | Myocardial necrosis marker increased* | 9 | 16.13 ( 8.37 - 31.08 ) | 16.12 ( 126.66 ) | 16 ( 8.3 ) | 4 ( 1.76 ) |
|  | Blood bilirubin abnormal | 5 | 14.98 ( 6.22 - 36.12 ) | 14.98 ( 64.76 ) | 14.88 ( 6.17 ) | 3.9 ( 0.98 ) |
|  | Haptoglobin decreased | 3 | 14.64 ( 4.7 - 45.58 ) | 14.64 ( 37.85 ) | 14.54 ( 4.67 ) | 3.86 ( 0.28 ) |
|  | Bilirubin conjugated increased | 7 | 14 ( 6.66 - 29.44 ) | 13.99 ( 83.89 ) | 13.91 ( 6.61 ) | 3.8 ( 1.39 ) |
|  | Blood alkaline phosphatase increased | 54 | 11.22 ( 8.59 - 14.67 ) | 11.19 ( 498.55 ) | 11.14 ( 8.52 ) | 3.48 ( 2.84 ) |
|  | Aspartate aminotransferase increased | 116 | 10.58 ( 8.81 - 12.7 ) | 10.51 ( 994.26 ) | 10.47 ( 8.72 ) | 3.39 ( 3.01 ) |
|  | Lipids increased* | 5 | 9.94 ( 4.13 - 23.94 ) | 9.94 ( 40 ) | 9.89 ( 4.11 ) | 3.31 ( 0.81 ) |
|  | Investigation abnormal | 4 | 8.73 ( 3.27 - 23.31 ) | 8.73 ( 27.26 ) | 8.7 ( 3.26 ) | 3.12 ( 0.48 ) |
|  | Carcinoembryonic antigen increased✝ | 5 | 8.46 ( 3.51 - 20.36 ) | 8.45 ( 32.73 ) | 8.42 ( 3.5 ) | 3.07 ( 0.73 ) |
|  | Blood chloride increased* | 3 | 8.1 ( 2.61 - 25.18 ) | 8.1 ( 18.6 ) | 8.07 ( 2.6 ) | 3.01 ( 0.1 ) |
|  | Transaminases increased | 47 | 7.74 ( 5.81 - 10.32 ) | 7.73 ( 274.26 ) | 7.7 ( 5.78 ) | 2.94 ( 2.34 ) |
|  | Blood lactate dehydrogenase increased* | 24 | 7.26 ( 4.86 - 10.84 ) | 7.25 ( 128.84 ) | 7.23 ( 4.84 ) | 2.85 ( 1.95 ) |
|  | Protein total decreased* | 6 | 7.06 ( 3.17 - 15.74 ) | 7.06 ( 31.09 ) | 7.04 ( 3.16 ) | 2.82 ( 0.82 ) |
|  | Blood creatinine increased | 109 | 6.76 ( 5.59 - 8.16 ) | 6.72 ( 529.53 ) | 6.7 ( 5.55 ) | 2.74 ( 2.4 ) |
|  | Blood uric acid increased | 10 | 6.63 ( 3.56 - 12.34 ) | 6.63 ( 47.65 ) | 6.61 ( 3.55 ) | 2.72 ( 1.26 ) |
|  | Alanine aminotransferase increased | 85 | 6.33 ( 5.11 - 7.84 ) | 6.31 ( 378.61 ) | 6.29 ( 5.08 ) | 2.65 ( 2.25 ) |
|  | Blood urea increased* | 16 | 5.28 ( 3.23 - 8.63 ) | 5.28 ( 55.36 ) | 5.27 ( 3.22 ) | 2.4 ( 1.37 ) |
|  | Heart rate decreased | 53 | 5.12 ( 3.91 - 6.71 ) | 5.11 ( 174.76 ) | 5.1 ( 3.89 ) | 2.35 ( 1.85 ) |
|  | Liver function test increased | 42 | 5.03 ( 3.72 - 6.82 ) | 5.02 ( 135.14 ) | 5.02 ( 3.7 ) | 2.33 ( 1.76 ) |
|  | Renal function test abnormal | 7 | 5 ( 2.38 - 10.51 ) | 5 ( 22.37 ) | 4.99 ( 2.38 ) | 2.32 ( 0.71 ) |
|  | Haematocrit decreased* | 17 | 4 ( 2.48 - 6.43 ) | 3.99 ( 38.09 ) | 3.99 ( 2.48 ) | 2 ( 1.09 ) |
| **Supplementary Table 3 (continued)** | | | | | | |
| System Organ Class(SOC) | Preferred term(PT) | Report number | ROR(95%Cl) | PRR(χ²) | EBGM(EBGM05) | IC(IC025) |
| Investigations | Red blood cell count decreased | 31 | 3.92 ( 2.75 - 5.57 ) | 3.91 ( 67.09 ) | 3.91 ( 2.75 ) | 1.97 ( 1.33 ) |
|  | Glomerular filtration rate decreased | 14 | 3.73 ( 2.21 - 6.3 ) | 3.73 ( 27.91 ) | 3.72 ( 2.2 ) | 1.9 ( 0.91 ) |
|  | Weight increased | 214 | 3.52 ( 3.08 - 4.03 ) | 3.49 ( 381.35 ) | 3.49 ( 3.05 ) | 1.8 ( 1.59 ) |
|  | Hepatic enzyme increased | 67 | 3.5 ( 2.75 - 4.45 ) | 3.49 ( 118.93 ) | 3.49 ( 2.74 ) | 1.8 ( 1.4 ) |
|  | Haemoglobin decreased | 67 | 2.58 ( 2.03 - 3.28 ) | 2.57 ( 64.38 ) | 2.57 ( 2.02 ) | 1.36 ( 0.98 ) |
| Nervous system disorders | Intracranial calcification* | 3 | 49.99 ( 15.9 - 157.15 ) | 49.98 ( 140.57 ) | 48.81 ( 15.53 ) | 5.61 ( 0.45 ) |
|  | Central nervous system necrosis✝ | 3 | 47.3 ( 15.06 - 148.58 ) | 47.29 ( 132.86 ) | 46.24 ( 14.72 ) | 5.53 ( 0.44 ) |
|  | Intracranial tumour haemorrhage✝ | 4 | 40.59 ( 15.08 - 109.2 ) | 40.58 ( 151.41 ) | 39.81 ( 14.8 ) | 5.32 ( 0.88 ) |
|  | Intracranial mass✝ | 4 | 10.22 ( 3.83 - 27.31 ) | 10.22 ( 33.11 ) | 10.17 ( 3.81 ) | 3.35 ( 0.55 ) |
|  | Cerebral disorder✝ | 19 | 7.6 ( 4.84 - 11.93 ) | 7.59 ( 108.35 ) | 7.57 ( 4.82 ) | 2.92 ( 1.86 ) |
|  | Vocal cord paralysis✝ | 3 | 6.6 ( 2.13 - 20.52 ) | 6.6 ( 14.22 ) | 6.59 ( 2.12 ) | 2.72 ( 0.01 ) |
|  | Brain oedema✝ | 17 | 5.51 ( 3.42 - 8.86 ) | 5.5 ( 62.45 ) | 5.49 ( 3.41 ) | 2.46 ( 1.45 ) |
|  | Taste disorder | 41 | 5.38 ( 3.96 - 7.32 ) | 5.37 ( 145.67 ) | 5.36 ( 3.95 ) | 2.42 ( 1.83 ) |
| Injury, poisoning and procedural complications | Radiation necrosis✝ | 4 | 22.52 ( 8.41 - 60.34 ) | 22.52 ( 81.36 ) | 22.28 ( 8.32 ) | 4.48 ( 0.78 ) |
|  | Radiation injury✝ | 4 | 15.5 ( 5.79 - 41.45 ) | 15.49 ( 53.83 ) | 15.39 ( 5.75 ) | 3.94 ( 0.69 ) |
|  | Peripheral nerve injury* | 3 | 11.64 ( 3.74 - 36.23 ) | 11.64 ( 29.02 ) | 11.58 ( 3.72 ) | 3.53 ( 0.22 ) |
|  | Sunburn | 17 | 8.82 ( 5.48 - 14.21 ) | 8.81 ( 117.25 ) | 8.78 ( 5.45 ) | 3.13 ( 1.93 ) |
| Musculoskeletal and connective tissue disorders | Muscle fatigue* | 19 | 16.09 ( 10.24 - 25.27 ) | 16.07 ( 266.42 ) | 15.95 ( 10.15 ) | 4 ( 2.54 ) |
|  | Sacral pain | 3 | 14.6 ( 4.69 - 45.47 ) | 14.6 ( 37.74 ) | 14.51 ( 4.66 ) | 3.86 ( 0.28 ) |
|  | Bone lesion✝ | 5 | 5.62 ( 2.34 - 13.53 ) | 5.62 ( 18.96 ) | 5.61 ( 2.33 ) | 2.49 ( 0.49 ) |
|  | Myalgia | 228 | 5.18 ( 4.54 - 5.9 ) | 5.12 ( 756.14 ) | 5.11 ( 4.48 ) | 2.35 ( 2.14 ) |
|  | Myositis* | 11 | 4.78 ( 2.65 - 8.65 ) | 4.78 ( 32.83 ) | 4.77 ( 2.64 ) | 2.25 ( 1.03 ) |
| Psychiatric disorders | Reading disorder✝ | 5 | 7.72 ( 3.21 - 18.57 ) | 7.72 ( 29.12 ) | 7.69 ( 3.2 ) | 2.94 ( 0.68 ) |
| **Supplementary Table 3 (continued)** | | | | | | |
| System Organ Class(SOC) | Preferred term(PT) | Report number | ROR(95%Cl) | PRR(χ²) | EBGM(EBGM05) | IC(IC025) |
| Respiratory, thoracic and mediastinal disorders | Hydrothorax* | 11 | 43.13 ( 23.73 - 78.37 ) | 43.1 ( 443.04 ) | 42.23 ( 23.24 ) | 5.4 ( 2.41 ) |
|  | Malignant pleural effusion✝ | 5 | 11.32 ( 4.7 - 27.28 ) | 11.32 ( 46.79 ) | 11.26 ( 4.68 ) | 3.49 ( 0.87 ) |
|  | Pneumonitis | 80 | 9.75 ( 7.82 - 12.15 ) | 9.71 ( 622.39 ) | 9.67 ( 7.76 ) | 3.27 ( 2.8 ) |
|  | Pulmonary toxicity | 20 | 9.56 ( 6.16 - 14.83 ) | 9.55 ( 152.38 ) | 9.51 ( 6.13 ) | 3.25 ( 2.13 ) |
|  | Pleural effusion* | 104 | 6.78 ( 5.59 - 8.23 ) | 6.75 ( 508.22 ) | 6.73 ( 5.55 ) | 2.75 ( 2.39 ) |
|  | Lung opacity | 6 | 6.16 ( 2.77 - 13.74 ) | 6.16 ( 25.87 ) | 6.15 ( 2.76 ) | 2.62 ( 0.73 ) |
|  | Pulmonary oedema* | 61 | 5.36 ( 4.17 - 6.89 ) | 5.34 ( 214.92 ) | 5.33 ( 4.15 ) | 2.41 ( 1.95 ) |
|  | Interstitial lung disease | 50 | 3.69 ( 2.8 - 4.88 ) | 3.69 ( 97.73 ) | 3.68 ( 2.79 ) | 1.88 ( 1.4 ) |
|  | Haemoptysis✝ | 26 | 3.47 ( 2.36 - 5.1 ) | 3.47 ( 45.6 ) | 3.46 ( 2.36 ) | 1.79 ( 1.11 ) |
| Skin and subcutaneous tissue disorders | Photosensitivity reaction | 73 | 16.21 ( 12.87 - 20.42 ) | 16.15 ( 1029.57 ) | 16.03 ( 12.73 ) | 4 ( 3.4 ) |
|  | Erythema multiforme* | 16 | 7.35 ( 4.5 - 12.01 ) | 7.34 ( 87.34 ) | 7.32 ( 4.48 ) | 2.87 ( 1.71 ) |
|  | Skin wrinkling* | 5 | 6.26 ( 2.6 - 15.05 ) | 6.26 ( 22.01 ) | 6.24 ( 2.59 ) | 2.64 ( 0.56 ) |
|  | Onychoclasis* | 9 | 4 ( 2.08 - 7.69 ) | 4 ( 20.19 ) | 3.99 ( 2.08 ) | 2 ( 0.71 ) |
|  | Toxic epidermal necrolysis* | 14 | 3.68 ( 2.18 - 6.22 ) | 3.68 ( 27.26 ) | 3.67 ( 2.17 ) | 1.88 ( 0.89 ) |
| General disorders and administration site conditions | Oedema | 154 | 11.47 ( 9.78 - 13.45 ) | 11.38 ( 1450.91 ) | 11.32 ( 9.66 ) | 3.5 ( 3.17 ) |
|  | Disease progression✝ | 290 | 8.81 ( 7.84 - 9.9 ) | 8.68 ( 1966.64 ) | 8.65 ( 7.7 ) | 3.11 ( 2.9 ) |
|  | Oedema peripheral | 154 | 6.69 ( 5.71 - 7.84 ) | 6.64 ( 736.16 ) | 6.62 ( 5.65 ) | 2.73 ( 2.44 ) |
|  | Drug resistance✝ | 55 | 6.18 ( 4.74 - 8.05 ) | 6.16 ( 237.14 ) | 6.14 ( 4.71 ) | 2.62 ( 2.11 ) |
|  | Face oedema | 19 | 4.88 ( 3.11 - 7.66 ) | 4.88 ( 58.45 ) | 4.87 ( 3.1 ) | 2.28 ( 1.38 ) |
|  | Generalised oedema | 11 | 3.83 ( 2.12 - 6.92 ) | 3.83 ( 22.97 ) | 3.82 ( 2.12 ) | 1.94 ( 0.8 ) |
|  | Peripheral swelling | 169 | 3.07 ( 2.64 - 3.57 ) | 3.05 ( 233.23 ) | 3.05 ( 2.62 ) | 1.61 ( 1.37 ) |
|  | Fatigue | 549 | 2.42 ( 2.22 - 2.63 ) | 2.38 ( 442.66 ) | 2.37 ( 2.18 ) | 1.25 ( 1.12 ) |
| **Supplementary Table 3 (continued)** | | | | | | |
| System Organ Class(SOC) | Preferred term(PT) | Report number | ROR(95%Cl) | PRR(χ²) | EBGM(EBGM05) | IC(IC025) |
| General disorders and administration site conditions | Death✝ | 602 | 2.41 ( 2.22 - 2.62 ) | 2.36 ( 479.93 ) | 2.36 ( 2.18 ) | 1.24 ( 1.12 ) |
| Gastrointestinal disorders | Defaecation disorder | 3 | 11.78 ( 3.79 - 36.64 ) | 11.78 ( 29.42 ) | 11.72 ( 3.77 ) | 3.55 ( 0.22 ) |
|  | Constipation | 463 | 7.68 ( 7 - 8.42 ) | 7.5 ( 2609.18 ) | 7.48 ( 6.82 ) | 2.9 ( 2.75 ) |
|  | Oesophageal stenosis✝ | 4 | 5.87 ( 2.2 - 15.67 ) | 5.87 ( 16.12 ) | 5.86 ( 2.2 ) | 2.55 ( 0.28 ) |
|  | Dysphagia✝ | 69 | 2.76 ( 2.18 - 3.5 ) | 2.75 ( 77.03 ) | 2.75 ( 2.17 ) | 1.46 ( 1.08 ) |
| Cardiac disorders | Sinus bradycardia | 20 | 8.5 ( 5.48 - 13.19 ) | 8.49 ( 131.6 ) | 8.46 ( 5.45 ) | 3.08 ( 2.01 ) |
|  | Sinus node dysfunction | 5 | 7.72 ( 3.21 - 18.57 ) | 7.72 ( 29.12 ) | 7.69 ( 3.2 ) | 2.94 ( 0.68 ) |
|  | Pericardial effusion* | 46 | 7.69 ( 5.75 - 10.27 ) | 7.67 ( 265.82 ) | 7.64 ( 5.72 ) | 2.93 ( 2.32 ) |
|  | Bradycardia | 90 | 6.55 ( 5.32 - 8.05 ) | 6.52 ( 419.34 ) | 6.5 ( 5.28 ) | 2.7 ( 2.31 ) |
|  | Atrioventricular block first degree* | 5 | 5.29 ( 2.2 - 12.73 ) | 5.29 ( 17.35 ) | 5.28 ( 2.19 ) | 2.4 ( 0.44 ) |
|  | Cardiac tamponade* | 6 | 4.77 ( 2.14 - 10.63 ) | 4.77 ( 17.83 ) | 4.76 ( 2.14 ) | 2.25 ( 0.54 ) |
| Blood and lymphatic system disorders | Spur cell anaemia | 13 | 784.09 ( 413.73 - 1485.98 ) | 783.51 ( 7349.57 ) | 567.07 ( 299.22 ) | 9.15 ( 2.9 ) |
|  | Haemolytic anaemia | 46 | 22.45 ( 16.78 - 30.03 ) | 22.39 ( 929.95 ) | 22.16 ( 16.57 ) | 4.47 ( 3.51 ) |
|  | Haemolysis | 29 | 14.84 ( 10.3 - 21.39 ) | 14.82 ( 370.98 ) | 14.72 ( 10.21 ) | 3.88 ( 2.81 ) |
|  | Hilar lymphadenopathy✝ | 3 | 13.28 ( 4.27 - 41.33 ) | 13.28 ( 33.84 ) | 13.2 ( 4.24 ) | 3.72 ( 0.25 ) |
|  | Lymphadenopathy mediastinal✝ | 5 | 9.39 ( 3.9 - 22.62 ) | 9.39 ( 37.32 ) | 9.35 ( 3.88 ) | 3.23 ( 0.79 ) |
|  | Anaemia | 232 | 4.82 ( 4.24 - 5.49 ) | 4.77 ( 692.24 ) | 4.76 ( 4.18 ) | 2.25 ( 2.04 ) |

Abbreviation: ROR, reporting odds ratio; CI, confidence interval; PRR, proportional reporting ratio; **χ²**, chi-squared; EBGM, empirical Bayesian geometric mean; EBGM05, the lower limit of the 95% CI of EBGM; IC, information component; IC025, the lower limit of the 95% CI of the IC. * Unexpected adverse events associated with alectinib from FAERS database.✝Events may related to disease progression rather than adverse events associated with alectinib.
